# Temperature Management With Paracetamol in Acute Stroke Patients: Evidence From Randomized Controlled Trials

**DOI:** 10.3389/fneur.2018.00917

**Published:** 2018-11-20

**Authors:** Huawei Chen, Hui Qian, Zhiwei Gu, Majun Wang

**Affiliations:** ^1^Department of Neurosurgery, Shaoxing Central Hospital, Shaoxing, China; ^2^Department of Neurosurgery, Zhejiang Zuji People's Hospital, Shaoxing, China

**Keywords:** paracetamol therapy, acute stroke, body temperature, meta-analysis, clinical trials

## Abstract

Whether or not paracetamol can improve functional outcomes in patients with acute stroke has been examined in several clinical trials. The inconsistent results of these trials have caused great controversy regarding the need for further studies. In the present meta-analysis, we have aimed to address this controversy. The main databases (Medline, Embase, and Cochrane Library) were searched for randomized controlled trials involving the use of paracetamol in acute stroke patients. Pooled relative risks (RRs) or mean differences (MDs) and 95% confidence intervals (CIs) were calculated using a random-effects model. A total of 1,836 patients were pooled from four phase II and two phase III trials. The use of paracetamol resulted in a significant reduction in body temperature after 24 h (MD, −0.21; 95% CI, −0.28 to −0.13; *P* < 0.001) and mortality rate after 7–14 days (RR, 0.62; 95% CI, 0.41–0.93; *P* = 0.02) when compared with the placebo group; however, no effect of paracetamol was observed in the modified Rankin Scale score (RR, 1.07; 95% CI, 0.91–1.27; *P* = 0.40) or Barthel Index score (RR, 0.98; 95% CI, 0.91–1.06; *P* = 0.63) at 30 or 90 days. No significant differences were observed with respect to serious adverse events between the paracetamol and the placebo groups (*P* > 0.05). Subgroup analyses were performed to detect the source of the heterogeneity, which showed that ischemic stroke, serious condition at baseline, and late time-to-treatment had adverse impacts on the effect of paracetamol post stroke. In conclusion, temperature management with paracetamol in acute stroke patients is safe. Although paracetamol reduced the mortality rate in the early stage of stroke, it did not appear to affect long-term mortality and functional recovery. It should be noted that this conclusion is based on the results from studies of poor quality. A large clinical trial with a focus on early treatment of patients with acute stroke is warranted.

## Introduction

Hyperthermia during the first 24 h from stroke onset is associated with large infarct volumes, high case fatalities, and poor functional outcomes (1, 2). Notably, in the first 12 h, the odds of poor functional outcomes double for every degree increase in body temperature (1); however, current guidelines provide weak recommendations regarding how to manage body temperature of stroke patients. According to the Australian Acute Stroke Guidelines (3), it is recommended that stroke patients with a fever >37.5°C should be treated with paracetamol. The Canadian Guidelines (4) also indicate that antipyretic therapy is required for acute stroke patients with a temperature >37.5°C (Evidence Level B). The US Stroke Guidelines (5) recommend the administration of antipyretic drugs when the body temperature is >38.0°C. Finding an inexpensive, safe, simple to use, and universally available drug to reduce body temperature is a promising approach to improve functional outcomes after stroke.

Paracetamol is a safe and commonly used antipyretic drug that has been shown to inhibit prostaglandin production in the central nervous system (6). It causes few adverse events, except for potential hepatic toxicity (6). High-dose paracetamol has been reported to reduce body temperature in patients with acute stroke, but there is still some debate on whether or not such therapy affects functional recovery. Four phase II trials indicated that treatment with paracetamol reduced body temperature by nearly 0.3°C within 4 h after the start of treatment (7–10) but showed no superiority in improving functional recovery (8, 10). These trials provided limited evidence due to the small samples and the purpose of testing safety and feasibility. The Paracetamol [Acetaminophen] in Stroke (PAIS) trial was a phase III study that enrolled 1,400 patients to determine the effect of high-dose paracetamol on the functional outcome (11). Paracetamol was observed to be associated with improved outcomes in a subgroup of patients with a body temperature ≥37.0°C (11); however, the PAIS trial was a *post-hoc* analysis with a weak level of evidence. Subsequently, the PAIS-2 trial was conducted to confirm the PAIS trial findings (12). Unfortunately, the trial was terminated early because of low patient recruitment (12). The results of the PAIS-2 trial showed that paracetamol did not improve functional outcome at 3 months (12). Noubiap and Kamtchum-Tatuene concluded that it would be a substantial waste of resources to continue investigating the efficacy of paracetamol in acute stroke patients due to the weak effect on functional recovery and difficulties in recruiting patients (13, 14). The Quality in Acute Stroke Care (QASC) trial (15) investigated the effect of a multidisciplinary intervention in targeting evidence-based management of fever, hyperglycemia, and swallowing dysfunction on stroke recovery, and it was found that multidisciplinary management delivered better patient outcomes at 90 days after discharge from stroke units. Hence, whether or not paracetamol improves functional outcomes in patients with acute stroke remains controversial.

The present study aimed to perform a meta-analysis of all randomized controlled trials (RCTs) to identify potential factors that have an impact on the effect of paracetamol on functional outcomes in patients with acute strokes and to determine whether or not a large clinical trial of treatment with paracetamol for patients with acute stroke is warranted.

## Methods

We conducted this meta-analysis according to the Preferred Reporting Items for Systematic Reviews and Meta-Analyses (PRISMA) format guidelines (13).

### Data sources and searches

The primary databases (Medline, Embase, and the Cochrane database) were searched for eligible published articles up to May 31, 2018 using the combination of free text words and MeSH terms as follows: (a) “paracetamol” or “acetaminophen” and (b) “stroke” or “hemorrhagic stroke” or “ischemic stroke.” Additional articles were identified by manual search from the references of original studies or review articles involving this topic. This search process of eligible studies was performed by two independent authors (Huawei Chen and Hui Qian).

### Selection criteria

Two of the authors (Huawei Chen and Zhiwei Gu) independently selected the articles from the databases as mentioned above. Any discordance was settled by a senior author (Majun Wang). The following inclusion criteria were used: (1) paracetamol was used to lower the body temperature during treatment for stroke; (2) patients were administrated paracetamol within 24 h after stroke onset; (3) only RCTs were included in this study; (4) there were no overlapping subjects across publications; (5) the language of the eligible studies was Chinese or English; and (6) the primary aim of the study was to investigate the effect of paracetamol on reducing body temperature after an acute stroke. The exclusion criteria were as follows: (1) the study that did not meet the inclusion criteria and (2) reviews, editorials, clinical conferences, abstracts, case reports, comments, protocols, and congresses were not taken into consideration.

### Data extraction and quality assessment

Data of interest were extracted by two authors (Huawei Chen and Zhiwei Gu) as follows: (1) identity (authors, years, and countries); (2) patients included in each study (age and gender); (3) eligibility criteria (inclusion and exclusion criteria); (4) type of treatment (paracetamol, dose, duration of treatment, and follow-up period); and (5) outcomes (functional outcomes, body temperatures, mortality rate, and serious adverse events). Any discordance was settled by a senior author (Majun Wang).

Short-term outcomes were assessed by body temperature at 24 h post admission, change in body temperature during 24 h post admission, death at 7 or 14 days post admission, and serious adverse events at the time of discharge. Long-term outcomes were assessed by the modified Rankin Scale (mRS) and Barthel Index (BI) scale scores at 30 or 90 days.

### Bias assessment

Biases of the included trials were assessed by two independent authors (Huawei Chen and Hui Qian) using a 7-point quality control, as recommended by the Cochrane Handbook (16). The items included selection, performance, detection, attrition, reporting, and other potential biases. Each item was categorized as a high, low, or unclear risk.

### Statistical methods

Review Manager Version 5.0 software (The Cochrane Collaboration, Software Update, Oxford, UK) provided by the Cochrane Collaboration was used to conduct the statistical analysis in the present meta-analysis. The binary outcomes were assessed by using pooled relative risk (RR) in a random-effects model, along with the 95% confidence interval (CI). Continuous outcomes were assessed using the mean difference (MD) in a random-effects model, along with the 95% CI. The heterogeneity from each study was calculated by the chi-squared value test and inconsistency index (*I*^2^). Significant heterogeneity was identified as a value of *P* < 0.05 or *I*^2^ > 50% (GRADE Handbook, available from guidelinedevelopment.org/handbook). We performed subgroup analysis to find the source of heterogeneity.

## Results

### Included studies characteristics

**Figure 1:**
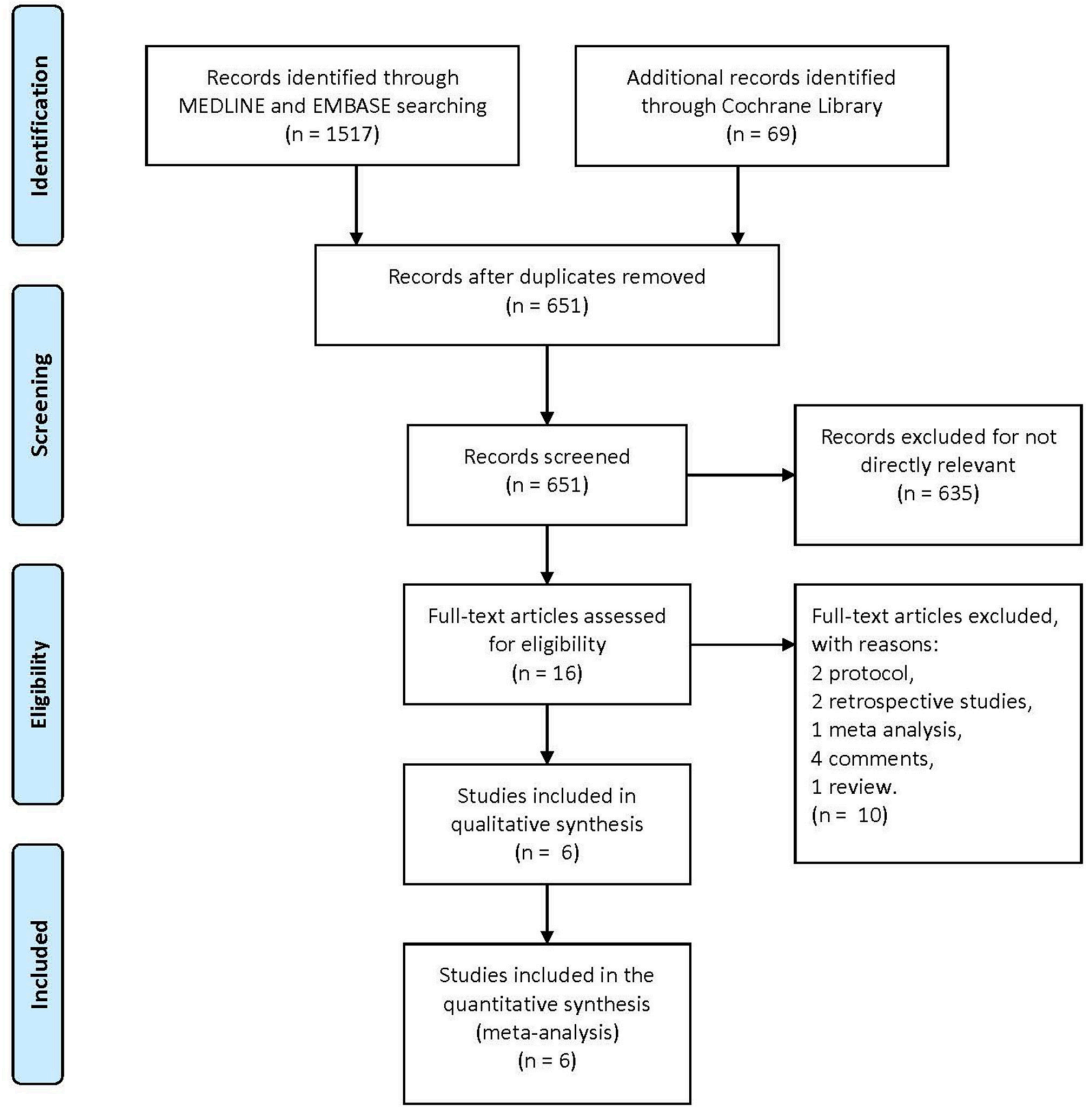
The study search, selection, and inclusion process.

A total of 1,586 articles were obtained from the three primary databases. After removing duplicates, the abstracts of 651 records were screened. Then, 16 full-text articles were assessed for eligibility after excluding 634 records due to a lack of relevance. Finally, two protocols, two retrospective studies, one meta-analysis, four comments, and one review were excluded because of the second exclusion criteria, and six RCTs were included in the quantitative synthesis (Figure 1). The characteristics of the included trials are summarized in Table 1.

**Table 1:** Characteristics of the included studies and outcome events.

**Trials**	**PAIS 2 2017**	**PAIS 2009**	**Diederik 2003**	**Scott 2001**	**Dippel 2001**	**Koennecke 2001**
**1. INFORMATION OF THE INCLUDED TRIALS**						
Regions	Multicenters in Netherlands	Multicenters in Netherlands	Three centers in Netherlands	Two centers in the USA	Three centers in Netherlands	A single center in Germany
Phases	III	III	II	II	II	II
Publication	Stroke	Lancet neurology	BMC cardiovascular disorders	Stroke	Stroke	Neurology
**2. ELIGIBILITY CRITERIA AND STUDY DESIGN**						
Inclusion criteria	Ischemic stroke or intracerebral hemorrhage, body temperature ≥ 36.5°C, age ≥ 18, treated within 12 h after stroke onset.	Ischemic stroke or intracerebral hemorrhage, body temperature between 36.5°C and 39.0°C, age ≥ 18, treated within 12 h after stroke onset.	Ischemic stroke in the anterior circulation, body temperature between 36.0°C and 39.0°C, treated within 24 h after stroke onset.	Ischemic or hemorrhagic stroke, body temperature < 38.5°C, NIHSS score ≥ 5, treated within 24 h after stroke onset.	Ischemic stroke in the anterior circulation, body temperature between 36.0°C and 39.0°C, treated within 24 h after stroke onset.	Ischemic stroke, body temperature < 37.5°C, treated within 24 h after stroke onset.
Exclusion criteria	Liver disease or alcohol abuse, allergy to paracetamol, abnormal liver enzymes, death appearing, and prestroke impairment interfering with the assessment of functional outcome.	Liver disease or alcohol abuse, allergy to paracetamol, abnormal liver enzymes, death appearing, and impairment before stroke that led to dependency.	Severe aphasia, using NSAID, allergy to paracetamol, liver failure or cirrhosis, renal failure, alcohol abuse, pregnancy, and impairment before stroke that led to dependency.	Infection, severe medical illnesses, allergy to paracetamol, placement of a urinary catheter, and participation in another interventional clinical trial for acute stroke.	Liver disease or alcohol abuse, allergy to paracetamol, using NSAID, and impairment before stroke that led to dependency.	Transient ischemic attack or hemorrhagic stroke, liver disease or alcohol abuse, and allergy to paracetamol.
Study design	Treated with high-dose paracetamol (6 g daily) or matching placebo for 3 days consecutively.	Treated with paracetamol (1 g daily, six times) f for 3 days consecutively.	Treated with paracetamol (1 g daily, six times) for 5 days consecutively.	Treated with paracetamol (650 mg daily, six times) g for 24 h.	Treated with paracetamol (0.5 or 1 g daily, six times) for 5 days consecutively.	Treated with paracetamol (1 g daily, four times) for 5 days consecutively.
**3. BASIC CHARACTERISTICS**						
Age Paracetamol	69	70	69	70	70	70
Placebo	69	70	65	67	68	71
Male Paracetamol	68 (50%)	374 (54%)	17 (65%)	9 (45%)	26 (51%)	9 (45%)
Placebo	76 (64%)	410 (58%)	16 (64%)	7 (37%)	16 (67%)	13 (59%)
NIHSS Paracetamol	6	6	18	14	9	5
Placebo	5	7	14	11	7	6
BT Paracetamol	36.9	36.9	37.2	36.9	36.9	36.6
Placebo	36.8	36.9	37.0	36.9	36.9	36.6
**4. OUTCOMES ASSESSMENTS**						
Primary outcomes	mRS score at 90 days	mRS score at 90 days	Body temperature at 24 h	Body temperature at 24 h	Body temperature at 24 h	Fever occurrence during the 5 days
Secondary outcomes	Body temperature at 24 h; unfavorable outcome, Barthel Index, and EuroQol-5D at 90 days	Body temperature at 24 h; Barthel Index, and serious adverse events at 14 days; unfavorable outcome, Barthel Index, and EuroQol-5D at 90 days	mRS score, Barthel Index, and serious adverse events at 30 days.	WBC counts, serious adverse events, and NIHSS at 24 and 48 h, and at 7 days.	Body temperature at 1 and 5 days, mRS score at 30 days, and serious adverse events during initial 7 days.	NIHSS and mRS on discharge.

*NIHSS, National Institutes of Health Stroke Scale; NSAID, Non Steroidal Anti Inflammatory Drugs; mRS, modified Rankin Scale; BT, Baseline Temperature*.

### Overall and sub-group analysis

**Figure 2:**
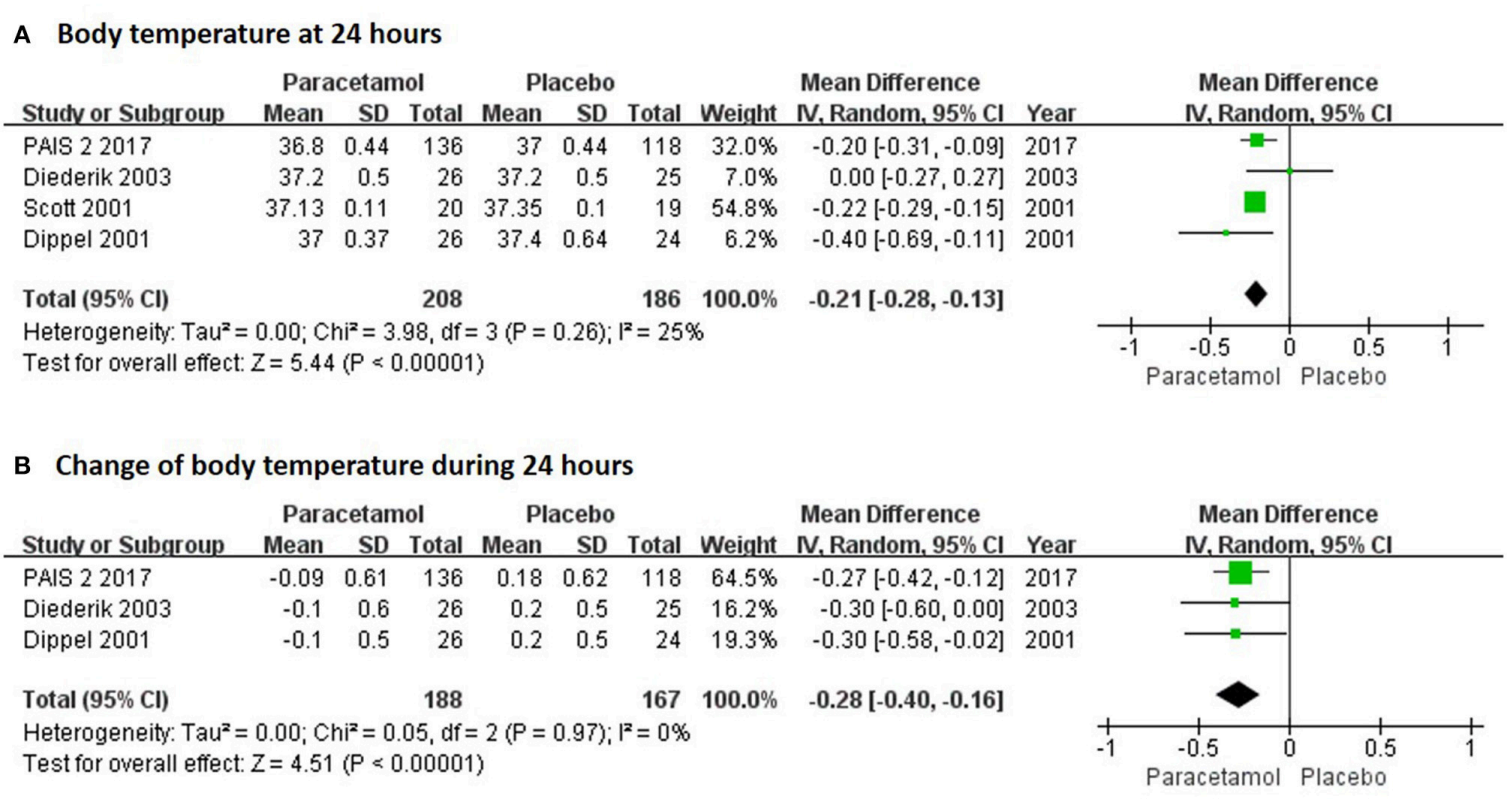
The pooled relative risk of the short-term efficacy outcomes. The diamond indicates the estimated relative risk (95% confidence interval) for all patients.

**Figure 3:**
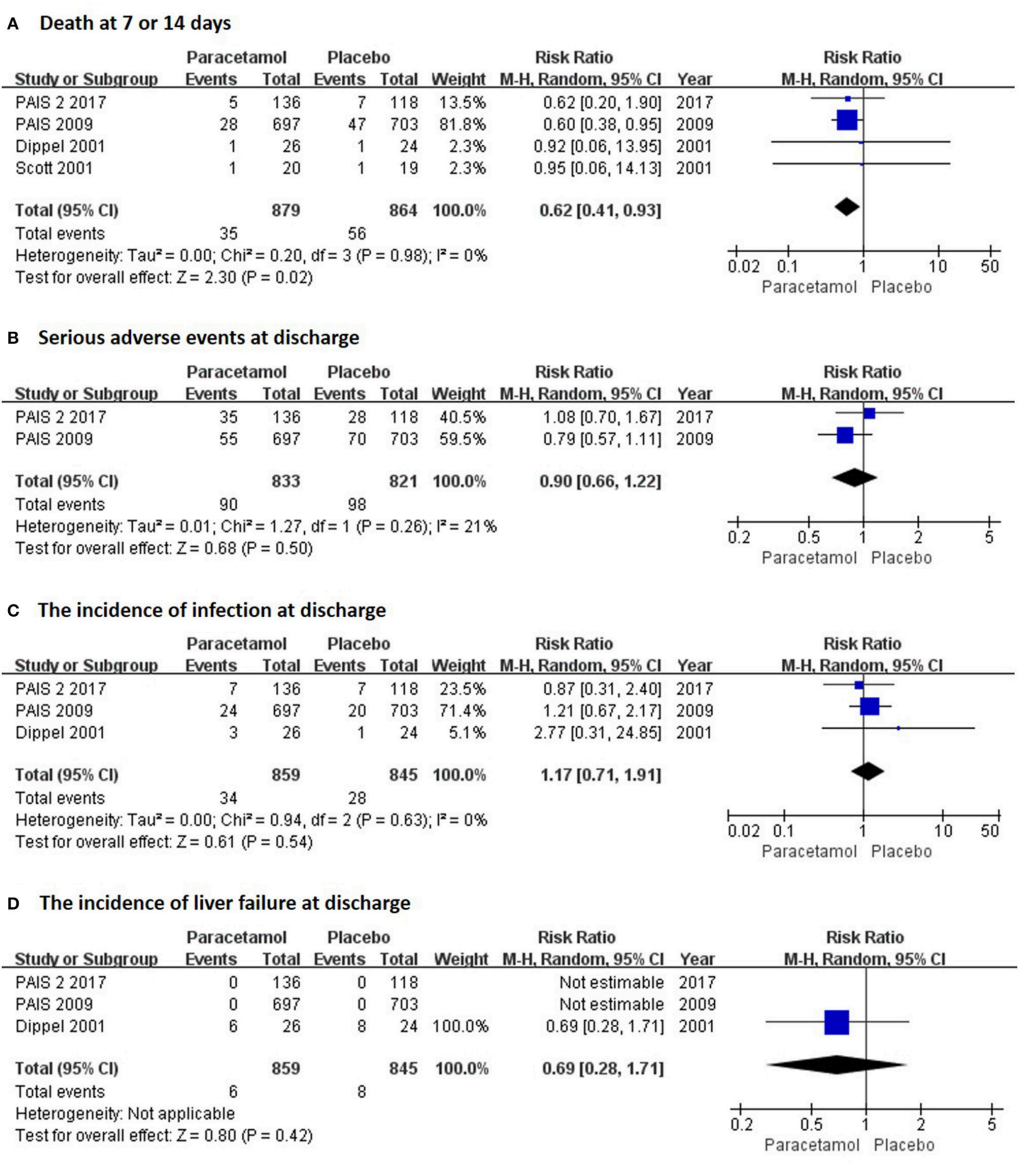
The pooled relative risk of the short-term safety outcomes. The diamond indicates the estimated relative risk (95% confidence interval) for all patients.

**Figure 4:**
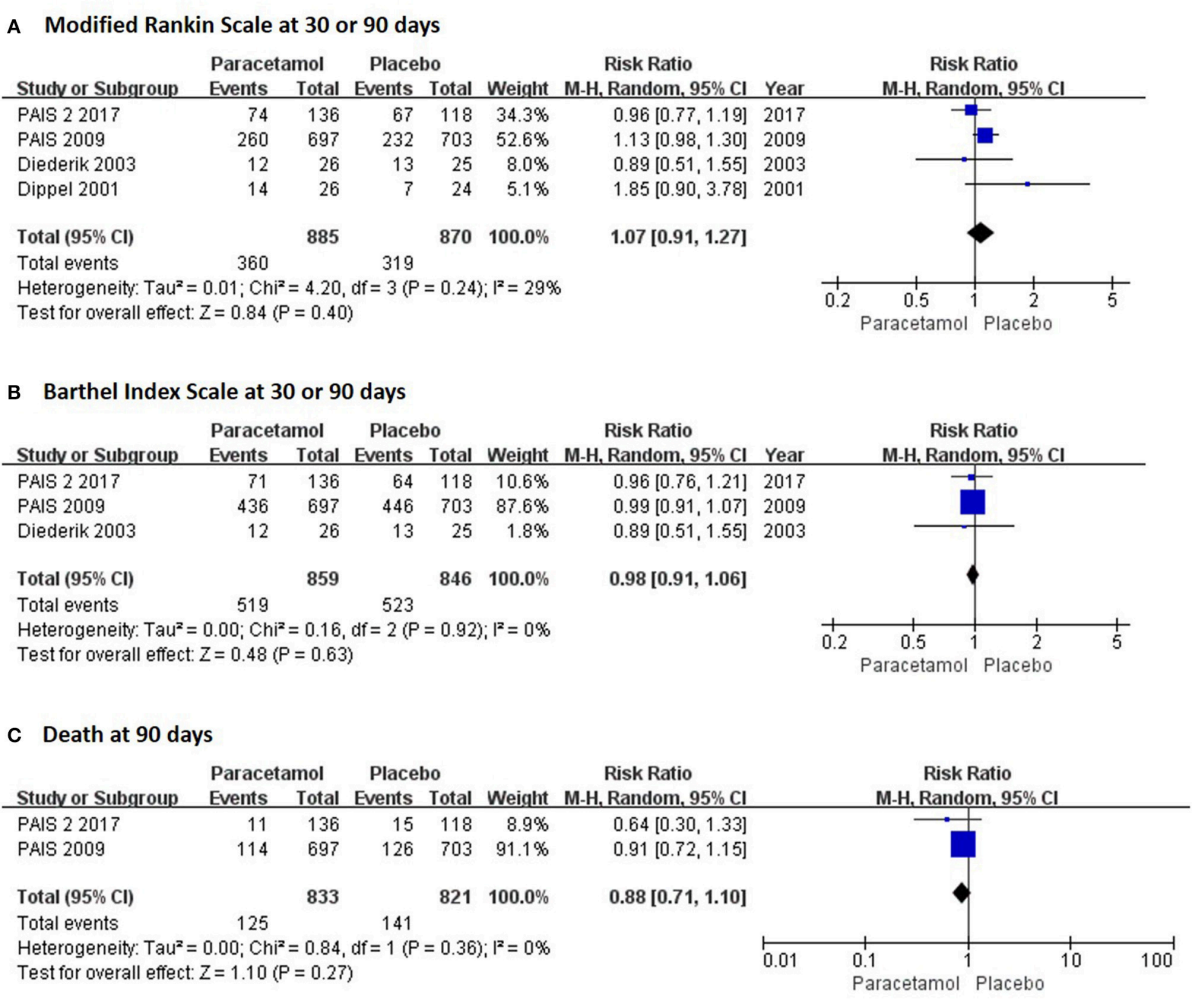
The pooled relative risk of the long-term efficacy outcomes. The diamond indicates the estimated relative risk (95% confidence interval) for all patients.

In the short term, paracetamol treatment showed a significant reduction of body temperature at 24 h post admission (MD, −0.21; 95% CI, −0.28 to −0.13; *P* < 0.001; Figure 2A), of a change in body temperature during 24 h post admission (MD, −0.28; 95% CI, −0.40 to −0.16; Figure 2B), and of mortality rates at 7 or 14 days post admission (RR, 0.62; 95% CI 0.41–0.93; *P* = 0.02; Figure 3A) when compared with the placebo injection. In addition, no significant difference was observed with respect to serious adverse events at the time of discharge (RR, 0.90; 95% CI, 0.66–1.22; *P* = 0.50; Figure 3B), including the incidence of infections (RR, 1.17; 95% CI, 0.71–1.91; *P* = 0.54; Figure 3C) and liver failure (RR, 0.69; 95% CI, 0.28–1.71; *P* = 0.42; Figure 3D). No significant heterogeneities were observed in any of the short-term outcomes (*I*^2^ < 30%; *P* > 0.05). In the long-term outcomes, paracetamol showed no effect based on the mRS (RR, 1.07; 95% CI, 0.91–1.27; *P* = 0.40; Figure 4A) or BI score (RR, 0.98; 95% CI, 0.91–1.06; *P* = 0.63; Figure 4B) at 30 or 90 days post stroke. In addition, there was no effect of paracetamol on 90-day mortality in the paracetamol group when compared with the placebo group (RR, 0.88; 95% CI, 0.71–1.10; *P* = 0.27; Figure 4C). No significant heterogeneities were observed in any of the long-term outcomes (*I*^2^ < 30%; *P* > 0.05). Sensitivity analysis indicated that all of the combined effect values were stable (Figure S1).

Subgroup analyses were performed to detect the source of heterogeneity. Long-term functional recovery was the main goal of paracetamol therapy but not all of the included trials provided long-term functional outcomes. Hence, we performed subgroup analysis and observed that paracetamol had no significant effect on mRS score in the 30- (RR, 1.24; 95% CI, 0.60–2.54; *P* = 0.56; Table 2) and 90-day follow-up subgroups (RR, 1.06; 95% CI, 0.91–1.25; *P* = 0.45; Table 2). In addition, several of the included trials recruited patients with ischemic or hemorrhagic stroke; however, there is presently insufficient evidence to support the clinical use of therapeutic hypothermia for hemorrhagic stroke, including intracerebral and subarachnoid hemorrhage (17). Therefore, we performed subgroup analyses of different types of stroke. We observed that paracetamol did not affect any outcomes, including body temperature at 24 h, mRS score at 30 or 90 days, mortality at 7 or 14 days, and infection rates at the time of discharge (*P* > 0.05;l Table 2) in patients with acute ischemic stroke; however, paracetamol reduced the body temperature at 24 h (MD, −0.21; 95% CI, −0.27 to −0.16; *P* < 0.001; Table 2) and mortality rate at 7 or 14 days (RR, 0.61; 95% CI, 0.40–0.93; *P* = 0.02; Table 2) in the ischemic and hemorrhagic stroke subgroups. Baseline stroke severity and the therapeutic time window were shown to be two major factors that influence the treatment of stroke patients. We found that paracetamol significantly reduced body temperature at 24 h and mortality rate at 7 or 14 days in patients with National Institute of Health Stroke Scale (NIHSS) score < 12, who developed therapeutic hypothermia within 12 h (*P* < 0.05; Table 2). The forest plots of the subgroup analyses are shown in Figures S2–S5.

**Table 2:** Subgroup analysis of outcomes.

	**Efficacy Outcomes**				**Safety Outcomes**			
	**Body temperature**		**Modified Rankin Scale**		**Death**		**Infection**	
	**MD (95% CI)**	***P-*value**	**RR (95% CI)**	***P*-value**	**RR (95% CI)**	***P*-value**	**RR (95% CI)**	***P*-value**
**1. FOLLOW-UP PERIOD**								
30 days	–	–	1.24 (0.60, 2.54)	0.56	–	–	–	–
90 days	–	–	1.06 (0.91, 1.25)	0.45	–	–	–	–
**2. TYPE OF STROKE**								
Ischemic	−0.20 (−0.59, 0.20)	0.33	1.24 (0.60, 2.54)	0.56	0.92 (0.06, 13.95)	0.95	2.77 (0.31, 24.85)	0.36
Ischemic and hemorrhagic	−0.21 (−0.27, −0.16)	< 0.001	1.06 (0.91, 1.25)	0.45	0.61 (0.40, 0.93)	0.02	1.11 (0.67, 1.85)	0.67
**3. STROKE SEVERITY AT BASELINE**								
***NIHSS** > **12***	−0.15 (−0.35, 0.05)	0.13	0.89 (0.51, 1.55)	0.68	0.95 (0.06, 14.13)	0.97	–	–
***NIHSS**<**12***	−0.25 (−0.42, −0.08)	0.004	1.10 (0.90, 1.34)	0.36	0.61 (0.40, 0.93)	0.02	1.17 (0.71, 1.91)	0.54
**4. TIME TO TREATMENT**								
Within 12 h	−0.20 (−0.31, −0.09)	0.0003	1.06 (0.91, 1.25)	0.45	0.60 (0.40, 0.92)	0.02	1.11 (0.67, 1.85)	0.67
Within 24 h	−0.21 (−0.37, −0.05)	0.01	1.24 (0.60, 2.54)	0.56	0.94 (0.14, 6.35)	0.95	2.77 (0.31, 24.85)	0.36

*MD, Mean Difference; CI, Confidence Intervals; RR, Relative Risks; NIHSS, National Institute of Health Stroke Scale*.

### Risk of bias

The risk of bias in the included studies is summarized in Figure S6. All of the included RCTs were double-blind trials, with the exception of the 2001 Scott trial (9). For the allocation concealment item, the 2001 Scott trial (9) had a high risk due to its open label design and the 2011 Koennecke trial (7) was unclear. For blinding of outcome assessment, the 2001 Scott trial (9) had a high risk and the 2003 Diederik trial (10) and 2001 Dippel trial (8) were unclear and were not mentioned in the studies. In addition, the three trials (7, 9, 10) showed high risks of incomplete outcome data for lack of serious adverse events.

## Discussion

The present meta-analysis focused on the efficacy and safety of paracetamol in reducing body temperature and improving functional outcomes in patients with stroke. We showed that paracetamol significantly reduced the body temperature 24 h after stroke onset. Moreover, paracetamol had excellent safety in reducing the mortality at 7 or 14 days and had no increase in serious adverse events at discharge; however, no superiority of paracetamol in improving functional recovery was observed, including mRS and BI scores at 30 or 90 days after stroke onset. Subgroup analyses showed that a high proportion of patients with ischemic stroke, serious condition at baseline, and late time-to-treatment had an adverse impact on the effects of paracetamol on body temperature at 24 h post admission and mortality rate at 7 or 14 days post admission; however, paracetamol did not affect functional outcomes in any subgroup.

Hyperthermia has been shown to be associated with more substantial ischemic neuronal injury and worse outcomes after stroke (18, 19). Paracetamol is regarded as an ideal solution to avoid hyperthermia because it is easily used, rapidly active, and widely available (6). The present study indicated that patients with paracetamol had a lower body temperature at 24 h when compared with the placebo group. Previous studies have reported that the odds of poor functional outcomes double for every degree increase in body temperature (1). Hence, the reduction of body temperature might aid significantly in recovery. However, the present study did not find any significant improvements of functional outcomes by using paracetamol in patients with acute ischemic stroke. In order to detect the source of heterogeneity, we performed subgroup analysis. We found that paracetamol significantly reduced body temperature at 24 h and decreased mortality at 7 or 14 days in patients with ischemic and hemorrhagic stroke. The PAIS trial also reported that more improvement in functional outcome was observed in hemorrhagic stroke patients than ischemic stroke patients after paracetamol therapy (11); however, previous studies provided insufficient evidence to support the clinical use of paracetamol therapy for hemorrhagic stroke, including intracerebral and subarachnoid hemorrhage (17). Hence, further studies are needed to determine whether or not paracetamol has effects on hemorrhagic stroke. In addition, we have also observed that paracetamol showed a significant therapeutic role in reducing body temperature at 24 h and mortality at 7 or 14 days in patients with a NIHSS score < 12 receiving therapy within 12 h. Previous studies have reported that peak body temperature is closely associated with stroke severity (20, 21). Paracetamol has no effect on reducing mortality at 7 or 14 days in this subtype of patients. Time-to-treatment is another important factor. Previous studies indicated that patients usually exhibit normothermia in the first 4 h of stroke. Peak temperature occurs 1.5–2 days after stroke (20). Notably, in the first 12 h of a stroke, the odds of poor functional outcome double for every degree increase in body temperature (1). Our study also indicated that the patients that received paracetamol within 12 h have a lower mortality rate at 7 or 14 days than patients who were administered paracetamol therapy within 24 h. Moreover, we also observed that paracetamol did not affect functional recovery in any subgroup of different stroke types, various stroke severities at baseline, and different time-to-treatment. Several issues might be involved in this ineffective attribute of paracetamol. First, none of the studies included in the present meta-analysis were of high quality. The four initial phase II trials had a small number of participants and were designed to test safety and feasibility (7–10). The PAIS trial was discontinued early after the inclusion of 1,400 patients despite a target of 2,500 because of a lack of funding (11). Also, the PAIS-2 trial was terminated early because of limited funding and difficulties in recruiting patients (12); the PAIS-2 trial only included 17% of the target sample size. Thus, the power of the study to detect a statistically significant effect for the original primary outcome measure of poor outcome was reduced (22). Second, the low completion rate in the fixed treatment period might be another relevant factor (14). In the 2003 Diederik trial, only 67% of the included patients completed the 5-day treatment. Similarly, 70% of the patients completed the full treatment period in the PAIS trial (11). Early complete recovery and death were the main reasons for not completing the treatment period.

Another consideration is the safety of paracetamol in patients with stroke. The present study indicated that paracetamol did not result in an increase in any serious adverse events, including liver failure and infections. In addition, paracetamol reduced the mortality rate in patients with acute stroke. No heterogeneity existed among these indices in the included trials. These results were consistent with previous studies that showed no side effects at therapeutic doses of paracetamol on cardiovascular and respiratory systems (6); however, it is thought that the 3-month follow-up period was too short to detect adverse events, especially a paracetamol-relevant liver disturbance (14).

Several limitations should be noted in the present meta-analysis. First, the included trials were not of high quality as mentioned above. Based on these unsatisfactory data, the results of the present meta-analysis were not sufficiently definitive to further inform clinical practice. Second, the various sample sizes of the included studies might result in an overwhelming weight in the meta-analysis. Third, meta-regression was not suitable to detect the influencing factors because of the limited included trials.

In conclusion, temperature management with paracetamol in acute stroke patients is safe. Although paracetamol could reduce the early mortality rate, paracetamol does not appear to affect long-term mortality and functional recovery. A large clinical trial of early treatment for patients with acute stroke is warranted.

## Author contributions

MW was the principal investigator. HC and HQ designed the study and developed the analysis plan. HC and ZG analyzed the data. HC contributed to writing the article. MW revised the manuscript and polished the language.

### Conflict of interest statement

The authors declare that the research was conducted in the absence of any commercial or financial relationships that could be construed as a potential conflict of interest.

## Supplementary material

Figure S1 Sensitivity analysis of all the outcomes, including body temperature at 24 h **(A)**, change in body temperature during 24 h **(B)**, death at 7 or 14 days **(C)**, serious adverse events at discharge **(D)**, the incidence of infections at discharge **(E)**, mRS score at 30 or 90 days **(F)**, Barthel Index scale at 30 or 90 days **(G)**, and death at 90 days **(H)**.

Figure S2 Subgroup analyses of follow-up period in modified Rankin Sacle score at 30 or 90 days.

Figure S3 Subgroup analyses of type of stroke in body temperature at 24 h **(A)**, modified Rankin Scale score at 30 or 90 days **(B)**, death at 7 or 14 days **(C)**, and the incidence of infections at discharge **(D)**.

Figure S4 Subgroup analyses of stroke severity at baseline in body temperature at 24 h **(A)**, modified Rankin Scale score at 30 or 90 days **(B)**, and death at 7 or 14 days **(C)**.

Figure S5 Subgroup analyses of time-to-treatment in body temperature at 24 h **(A)**, modified Rankin Scale score at 30 or 90 days **(B)**, death at 7 or 14 days **(C)**, and the incidence of infections at discharge **(D)**.

Figure S6 Risk of bias: a summary table for each risk of bias item for each study.
