# Supplementary figures and images for: Temperature Management With Paracetamol in Acute Stroke Patients: Evidence From Randomized Controlled Trials
Source: Front Neurol. 2018 Nov 20;9:917. doi: 10.3389/fneur.2018.00917 (PMC6255928; doi:10.3389/fneur.2018.00917)

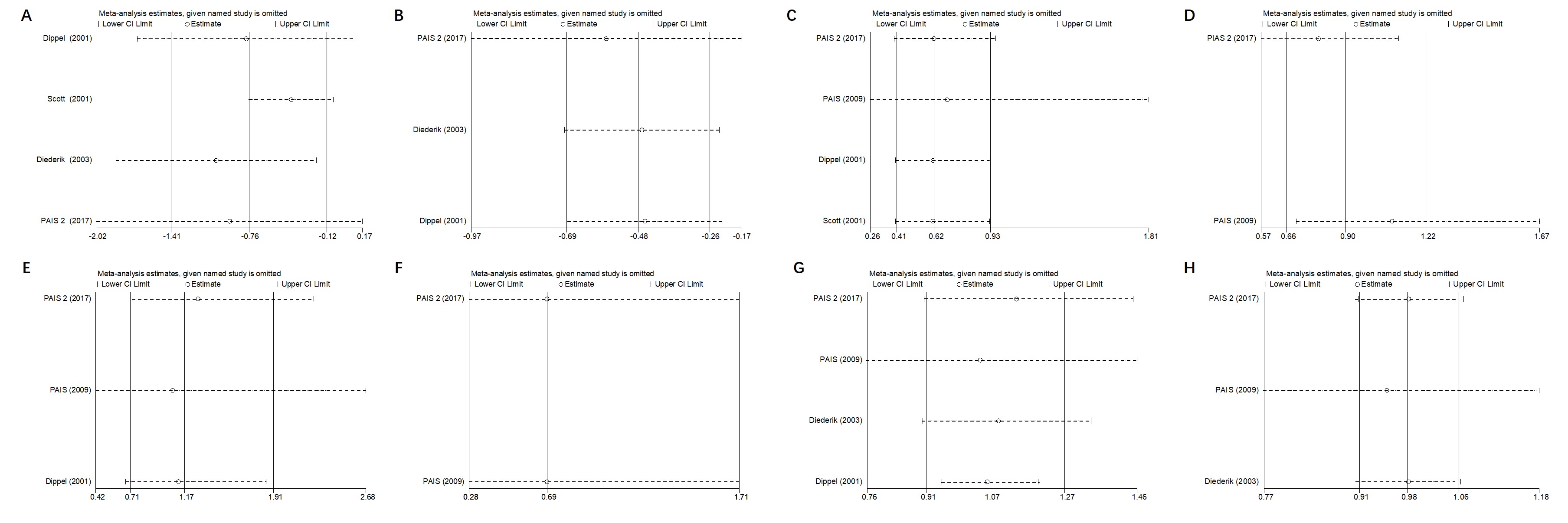

Supplement: Figure S1 — Sensitivity analysis of all the outcomes, including body temperature at 24 h (A), change in body temperature during 24 h (B), death at 7 or 14 days (C), serious adverse events at discharge (D), the incidence of infections at discharge (E), mRS score at 30 or 90 days (F), Barthel Index scale at 30 or 90 days (G), and death at 90 days (H). [file Image_1.JPEG]

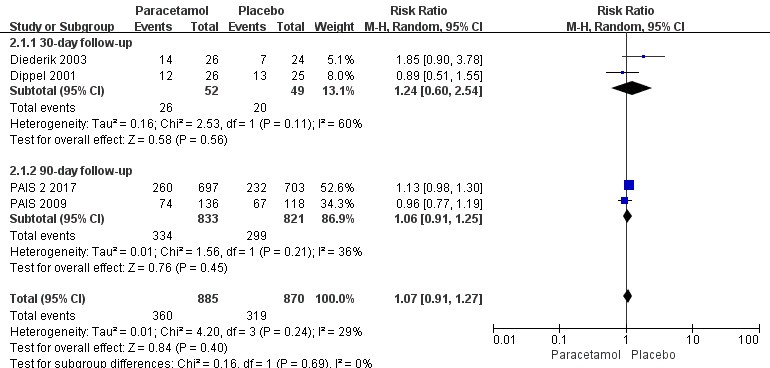

Supplement: Figure S2 — Subgroup analyses of follow-up period in modified Rankin Sacle score at 30 or 90 days. [file Image_2.JPEG]

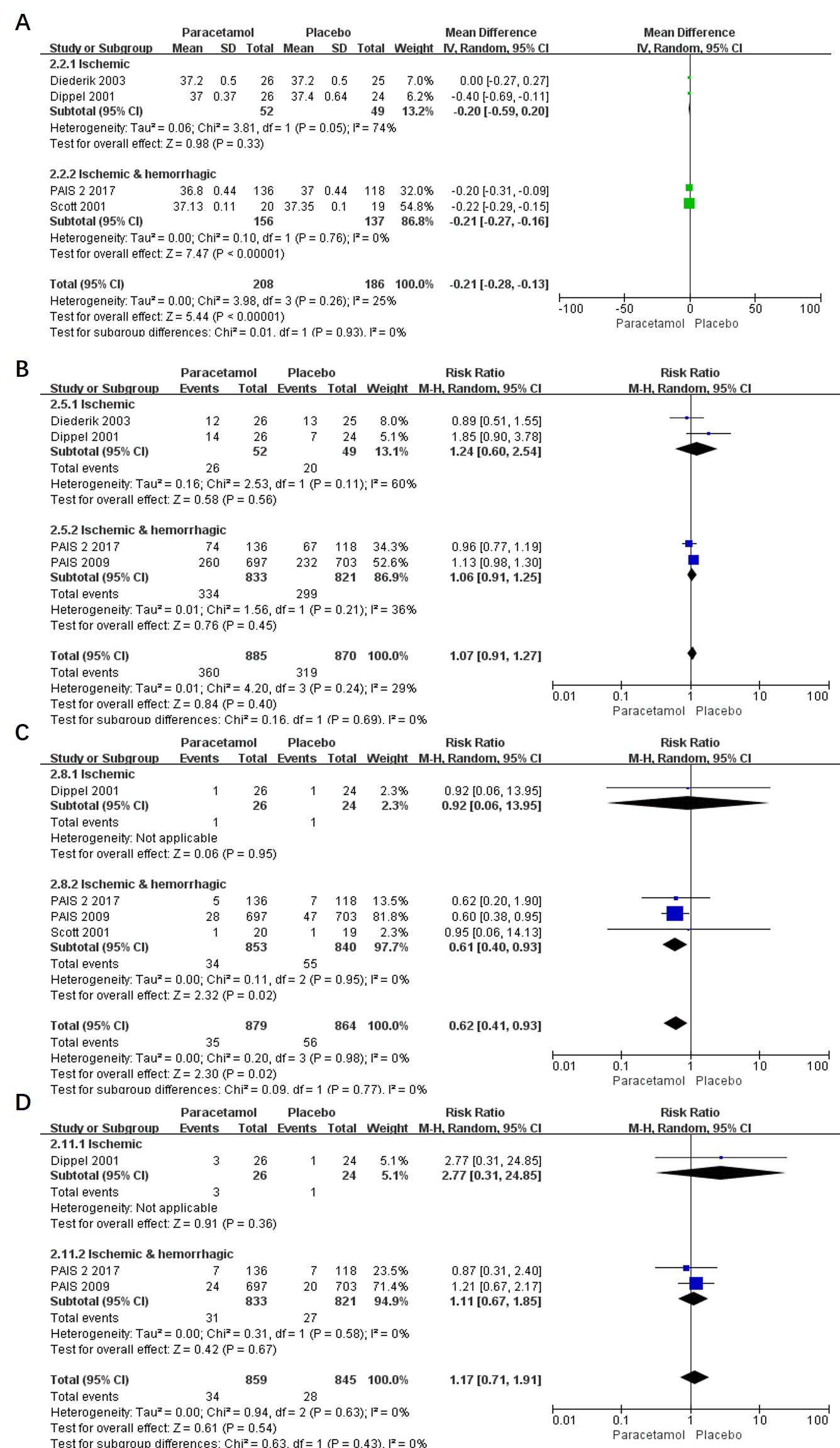

Supplement: Figure S3 — Subgroup analyses of type of stroke in body temperature at 24 h (A), modified Rankin Scale score at 30 or 90 days (B), death at 7 or 14 days (C), and the incidence of infections at discharge (D). [file Image_3.JPEG]

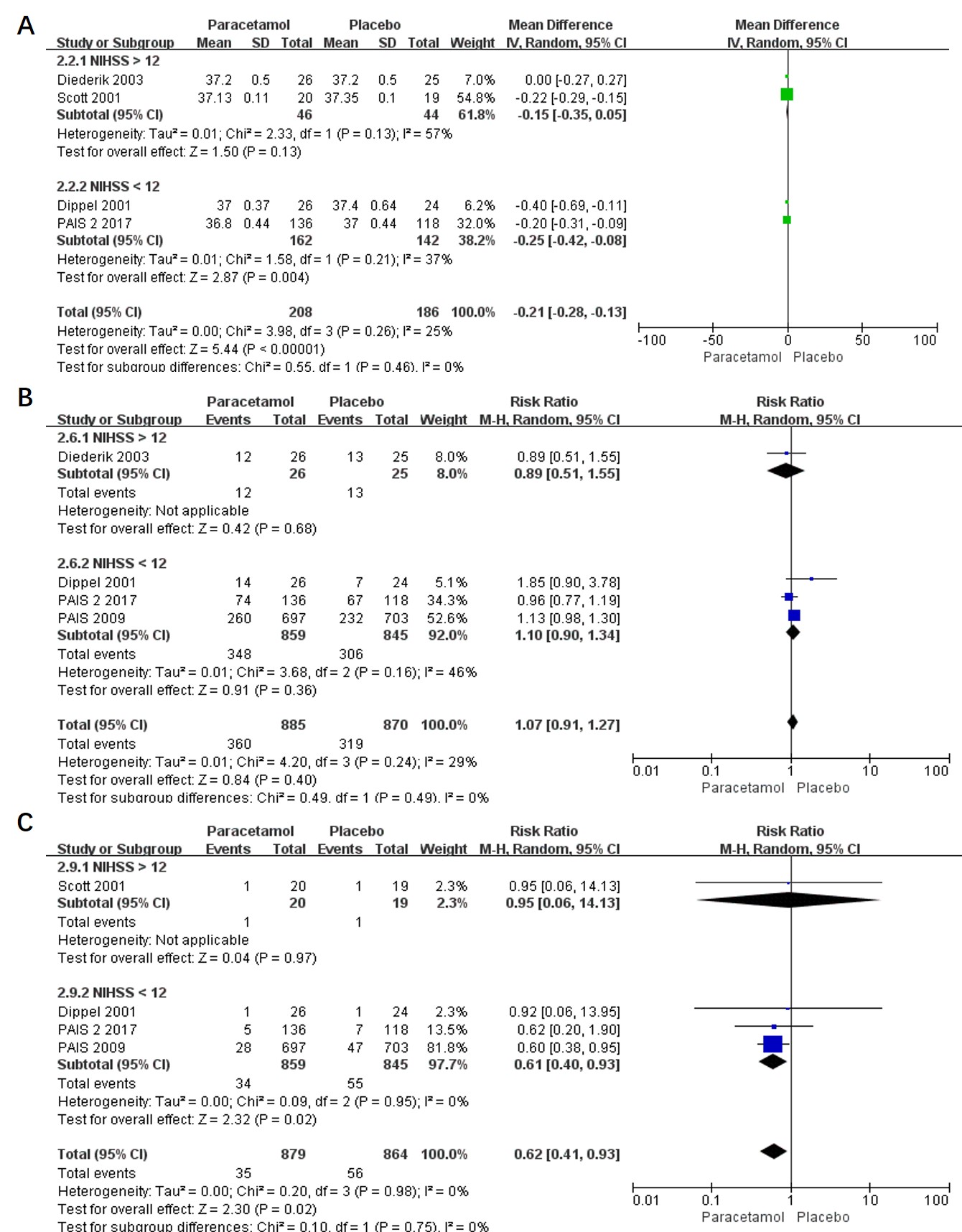

Supplement: Figure S4 — Subgroup analyses of stroke severity at baseline in body temperature at 24 h (A), modified Rankin Scale score at 30 or 90 days (B), and death at 7 or 14 days (C). [file Image_4.JPEG]

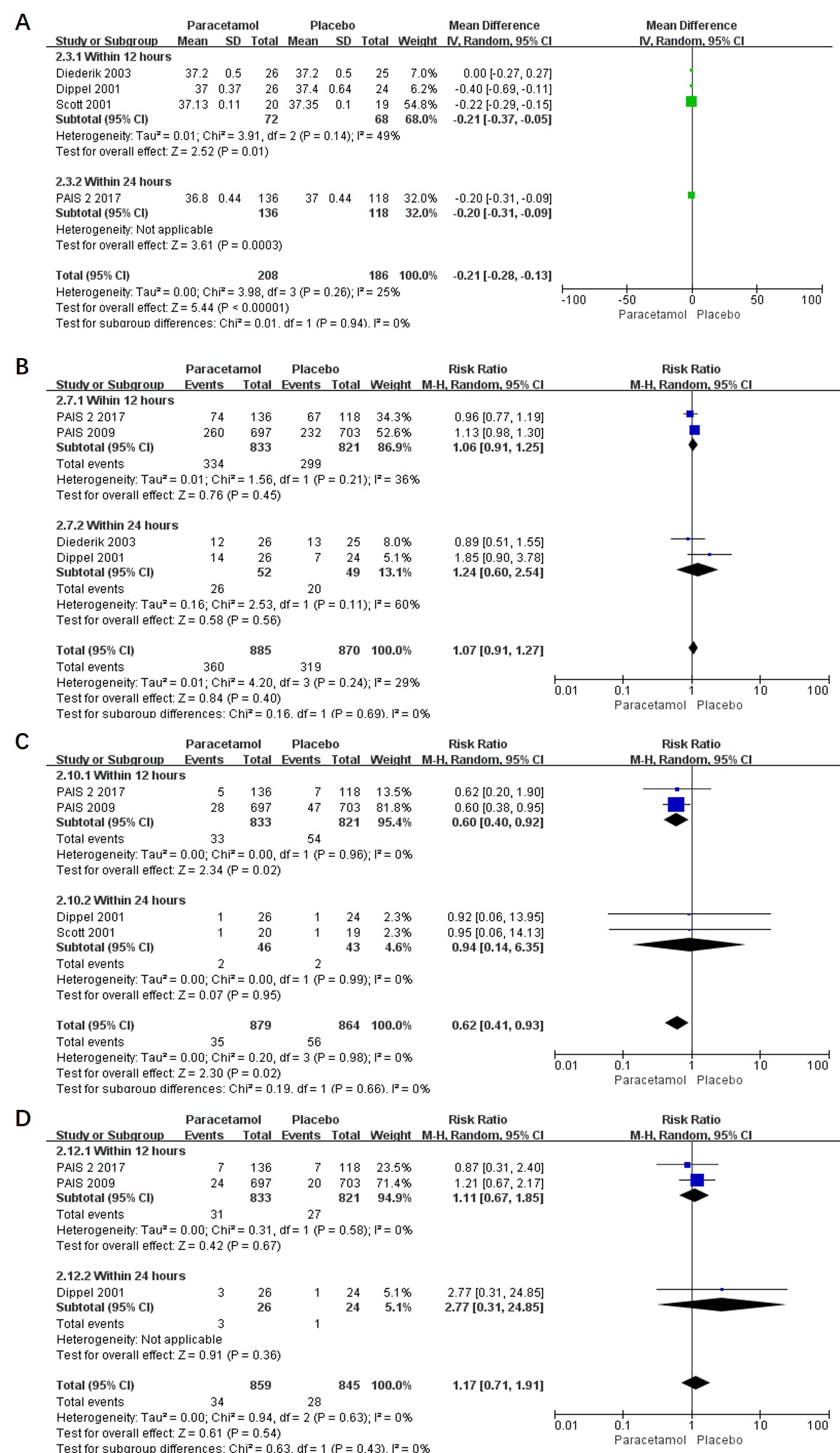

Supplement: Figure S5 — Subgroup analyses of time-to-treatment in body temperature at 24 h (A), modified Rankin Scale score at 30 or 90 days (B), death at 7 or 14 days (C), and the incidence of infections at discharge (D). [file Image_5.JPEG]

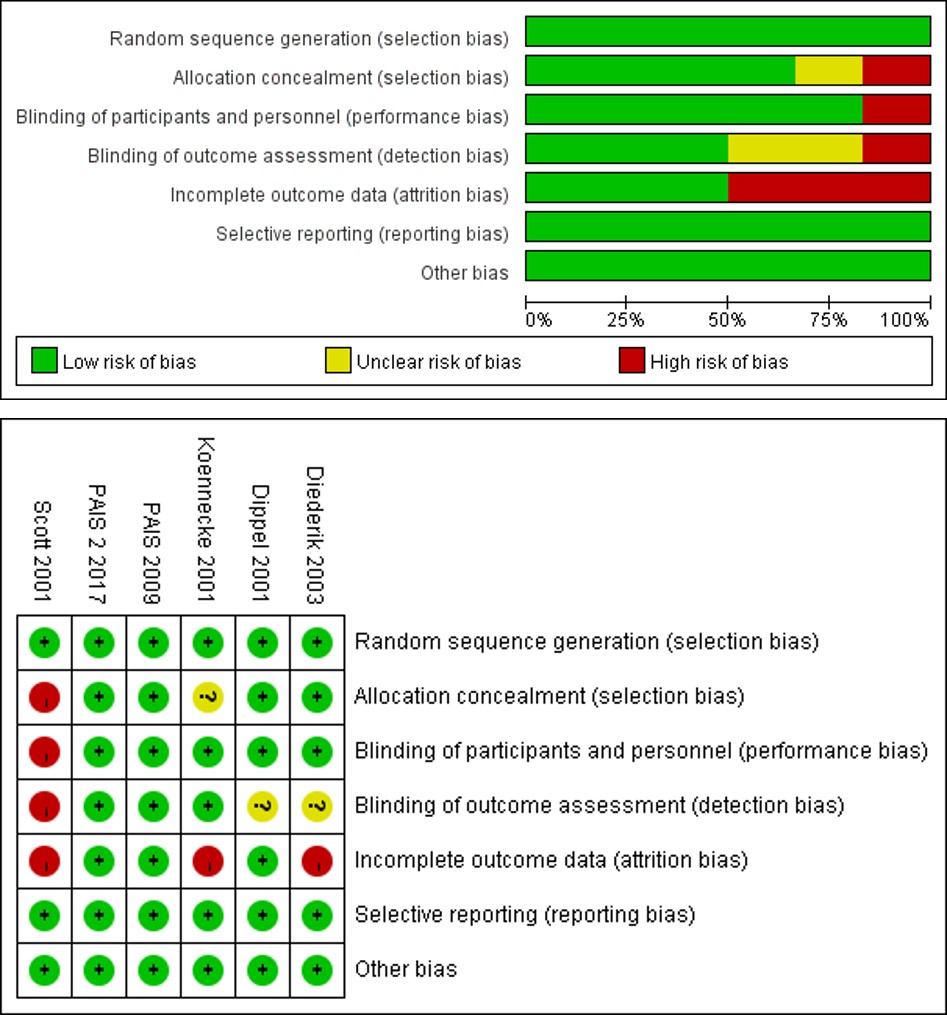

Supplement: Figure S6 — Risk of bias: a summary table for each risk of bias item for each study. [file Image_6.JPEG]
